# Modifications of Plasma Membrane Organization in Cancer Cells for Targeted Therapy

**DOI:** 10.3390/molecules26071850

**Published:** 2021-03-25

**Authors:** Anna Choromańska, Agnieszka Chwiłkowska, Julita Kulbacka, Dagmara Baczyńska, Nina Rembiałkowska, Anna Szewczyk, Olga Michel, Agnieszka Gajewska-Naryniecka, Dawid Przystupski, Jolanta Saczko

**Affiliations:** 1 Department of Molecular and Cellular Biology, Wroclaw Medical University, Borowska 211A, 50-556 Wroclaw, Poland; julita.kulbacka@umed.wroc.pl (J.K.); dagmara.baczynska@umed.wroc.pl (D.B.); nina.rembialkowska@umed.wroc.pl (N.R.); a.szewczyk@umed.wroc.pl (A.S.); michel.olga.maria@gmail.com (O.M.); agnieszka.gajewska-naryniecka@umed.wroc.pl (A.G.-N.); jolanta.saczko@umed.wroc.pl (J.S.); 2 Department of Paediatric Bone Marrow Transplantation, Oncology and Haematology, Wroclaw Medical University, Borowska 213, 50-556 Wroclaw, Poland; dawid.przystupski@student.umed.wroc.pl

**Keywords:** plasma membrane, cancer cells, targeted therapy, multidrug resistance, membrane receptors modulation, ion channel modulation, electroporation, sonoporation, microgravity

## Abstract

Modifications of the composition or organization of the cancer cell membrane seem to be a promising targeted therapy. This approach can significantly enhance drug uptake or intensify the response of cancer cells to chemotherapeutics. There are several methods enabling lipid bilayer modifications, e.g., pharmacological, physical, and mechanical. It is crucial to keep in mind the significance of drug resistance phenomenon, ion channel and specific receptor impact, and lipid bilayer organization in planning the cell membrane-targeted treatment. In this review, strategies based on cell membrane modulation or reorganization are presented as an alternative tool for future therapeutic protocols.

## 1. Introduction

Currently, researchers focus on the plasma membrane of cancer cells as a promising therapy target [1]. The cell membrane is a natural barrier that determines and regulates the transport of molecules in and out of the cytoplasm. It can be determined by the cell membrane composition, which is dependent on the content of various lipids (e.g., phosphatidylcholine, cholesterol, ceramides) [1,2,3] and proteins (e.g., channel proteins, receptor proteins, or functional proteins) [4]. Cell membrane organization also plays a role in cell signaling, redox balance, cell death pathways, or metastasis processes [1,5].

Mitigating or softening the lipid bilayer barrier can significantly affect the existing cell functions. However, we should pay attention to the differences in plasma membrane between normal and cancer cells. Basic ones include the shape, i.e., asymmetry, the composition, which is strongly determined by the cholesterol content and defines membrane permeability [3,6]. Rivel et al. showed that the cholesterol molar ratio change from 0% to 33% could reduce permeability [6]. It was also shown that the cancer cell membrane varies in the degree of unsaturation compared to normal cells [3]. Certain methods can affect, damage, or modify fluidity and elasticity of the cell membrane, i.e., pharmacological [7], physical (e.g., electroporation [8,9], or sonoporation [10], mechanical (e.g., microcentrifugation or microgravity) [11,12] or genetical [13,14,15]. The main dilemma is which method based on membrane-lipid therapies would be appropriate for various types of cancers. Each of these methods should be considered individually based on cancer type. This study demonstrates various methods that implicate the plasma membrane modifications, which can be a promising target of anticancer therapies. Moreover, this approach may stimulate the development of new therapeutics and treatment protocols in the future.

## 2. The Multidrug Resistance Phenomenon and Its Modulators

### 2.1. Types of Cancer Multidrug Resistance and Its Mechanisms

The multidrug resistance (MDR) phenomenon is one of the main challenges of successful oncological therapy [16]. It is a process in which cancer cells primarily sensitive to a single anticancer compound become resistant to many unrelated drugs that are functionally and/or structurally distinct. Cancer cells become resistant to many chemotherapeutic drugs that have different molecular targets [17]. MDR of cancer cells may result from their primary genetics, which is then called internal MDR. In that variant, cells present primary resistance to chemotherapy before exposure to the cytostatic drugs. The other type is an acquired MDR, which is the more problematic because it leads to ineffective therapies even when the drug concentration is increased to a toxic level [18]. However, not all cancers have the same probability of developing MDR. Its secondary form is prevalent in neoplasms of the kidneys, ovary, bladder, and lymphoblastic leukemia or small cell lung cancer [19]. Primary MDR is characteristic for tumors of the secretory organs such as the liver, colon, or adrenal glands [20]. Multidrug-resistant cells use a variety of defense mechanisms [21]. Most of the tumor MDR strategies involve proteins that have both structural and enzymatic functions. The mechanisms of MDR can be divided into extracellular (external) and cellular (internal). The external ones result from special conditions on the structural level, such as reduced permeability of blood vessels or tissue barriers that prevent drug molecules from penetrating target cells [22]. Heterogeneity is caused by different hydrostatic pressure in distinct parts of the tumor or differences in vascularization. Such heterogeneity may result in the formation of genetically different subpopulations of neoplastic cells, which predispose to significant differences in their response to the chemotherapeutic drug [23]. Internal MDR strategies take advantage of numerous aspects of cell function. In neoplastic diseases, the most common is the transport-dependent MDR, in which the cytostatic do not reach therapeutic concentrations in the cells. This form entails a reduced uptake of drugs from the external environment, resulting from changes in the cytoplasmic membrane structure, hence its permeability to xenobiotics. It also involves the cellular efflux phenomenon based on the active removal of therapeutic molecules from inside the cells via protein-membrane transporters. Difficulties can also occur during the intracellular transport of cytostatic between the nucleus and the cytoplasm. Other internal factors influencing the development of MDR in cancer cells include cell cycle disorders, drug binding in lysosomes or other intracellular organelles [24], drug inactivation by Phase I and Phase II enzymes [17], disruption of the pathways leading to apoptosis. An apoptosis process disturbance occurs via blocking the functionality of proapoptotic proteins and overexpression of proteins operating as programmed death inhibitors. The frequently observed lack of sensitivity to chemo- or radiotherapy is caused by overexpression of antiapoptotic proteins, including Bcl-2 family proteins or apoptosis inhibitors. The cause may also lie in (simultaneous or alternative to overexpression) underexpression of proapoptotic proteins, which also belong to the Bcl-2 family. Bcl-2 family proteins play an essential role in apoptosis regulation. The ratio of pro- and antiapoptotic proteins is the factor deciding about taking or avoiding the programmed cell death pathway. Mutations of the Bcl-2 family (protein p53, genes BAX, and BAK) are often observed in neoplastic cells, which promote the appearance of apoptotic resistance [25]. Another internal factor that can inhibit the action of anticancer drugs is the antioxidant defense system. Most chemotherapeutic agents induce free radical production, and oxidative stress causes damage at the level of proteins, nucleic acids, and cell membranes. Antioxidant mechanisms that protect cells from free radicals also protect cancer cells from free radical damage. These mechanisms are based on the activity of proteins such as superoxide dismutase, catalase, glutathione peroxidase, and cytochromes [17,26]. Usually, these enzymes are overexpressed in tumor cells [16].

### 2.2. Proteins Determining MDR

Most of the known proteins responsible for MDR belong to the ABC family (ATP-binding cassette family). There are 49 identified transporters representing the ABC family in the human proteome. The most important membrane proteins from that group include P-glycoprotein (P-gp/ABCB1), multidrug resistance-associated protein 1 (MRP1/ABCC1), multidrug resistance-associated protein 2 (MRP2/ABCC2), breast cancer resistance protein (BCRP/ABCG2) [16]. Their molecules contain two basic structural elements: the membrane-spanning domain (MSD) and located in the cytosol side, the ATP-binding domain, also known as nucleotide-binding folds (NBFs) [27]. The hydrophilic region of NBF is responsible for the binding and hydrolysis of ATP [28]. The energy released in ATP decomposition is used to transport substrates through extracellular and intracellular lipid membranes [29]. Membrane transporters in eukaryotic cells are exporters. They are responsible for removing harmful substances—xenobiotics and natural toxins—from the cytoplasm outside a cell or specific intracellular compartments. ABC transporters enable the functioning of numerous tissue barriers, but their increased expression is responsible for cancer cell resistance [30]. More than 50% of used anticancer drugs are removed from the cell by the active transport mediated by P-gp transporters. Therefore, there is a tendency to intensify the antitumor activity and decrease the chemotherapeutic systemic toxicity by inhibiting transport mediated by P-gp [31]. P-gp is composed of 1280 amino acids that form two homologous MSD containing six transmembrane domains and two ATP-binding regions, separated by a flexible linker. The secondary structure of the linker region is probably responsible for the coordinated functioning of both P-gp parts. It is responsible for the correct interaction of the two ATP binding domains. The NBFs are located in the cytoplasm and transfer the energy to transport the substrates across the membranes [32].

The xenobiotic removal mechanism by the P-gp has been described in several ways; however, the exact position of the substrate interaction with the protein is not precisely defined [33]. The three main models describing the P-gp mechanism of action are the pore formation model, the flippase model, and the hydrophobic vacuum cleaner model [34]. The last model assumes that P-gp recognizes substrates embedded in the inner layer of the cell membrane and transports them through a protein channel. These substrates are hydrophobic and have a positive charge at physiological pH. However, it was observed that uncharged hydrophilic substrates such as colchicine are also removed from cells by P-gp, suggesting a slightly different mechanism. Rosenberg et al. suggested that P-gp undergoes conformational changes in domains integral with the cell membrane during ATP binding, facilitating translocation of the P-gp substrate [35]. According to the pore formation model, after ATP binding, the transmembrane domains are reorganized along with the entire membrane depth. It creates a central pore through which hydrophobic substrates are removed [36]. Another hypothesis is that P-pg is a flippase that detects the xenobiotic inside the cell membrane and removes it into the extracellular space [31].

### 2.3. MDR Modulators

Based on the knowledge about the P-gp structure and mechanism of action, researchers have been identified substances that modulate the action of P-gp. Due to the action of these substances, the MDR of neoplastic cells can be manipulated to some extent to improve the effectiveness of anticancer therapies. Currently, there are three generations of P-gp inhibitors. First-generation inhibitors, including cyclosporin A, erythromycin, and verapamil [37], originally had a different therapeutic purpose. In their structure, the molecules show a remarkable similarity to the substrates of target transporters [38], and therefore, their competitive activity is inhibited. However, due to its low affinity for MDR proteins, the effectiveness of this generation of chemosensitizers requires their administration in high doses, which leads to severe toxicity [39]. Compounds such as valspodar, dexverapamil or gallopamil, belong to the group of second-generation inhibitors. These modulators have lower toxicity, a better pharmacological profile, but still a low affinity for P-gp [40]. Unfortunately, these substances lack specificity and are competitive inhibitors of cytochrome P450 3A4 (CYP3A4). As a result of these reactions, the metabolism of all xenobiotics present in the system is impaired, and the pharmacokinetic profile of the administered cytostatics is disturbed [41]. Failure of therapy with chemosensitizers of the first two groups led to the development of the third generation of MDR modulators. They are characterized by a high affinity for P-gp, which excludes the risk of being associated with other transporters and disrupting their functions. Moreover, when administered in small amounts, they bind to the target protein, forcing a conformational change of the transporter molecule, thus inhibiting its activity and the release of cytostatic molecules outside the tumor cell [42]. This highly promising group of inhibitors includes substances such as tariquidar, biricodar, annamycin, mitotane, and laniquidar [43].

Modulators of other proteins involved in MDR have also been developed. The MRP family proteins are inhibited by MS209 (dofequidar fumarate) [44] and BSO (buthionine sulfoximine), which directly block the formation of glutathione conjugates by GST [42]. In multidrug resistance resulting from cellular crypts and LRP, the pyridine derivative (PAK-104P) is used as a modulator [42]. BCRP inhibition enables elacridar, tariquidar, biricodar, which are P-gp inhibitors [45], as well as fumitremorgin C (FTC) and dietary herbal components [46]. Plant-derived substances can influence the modulation of P-gp activity and expression. Notably, some of them, by inhibiting P-gp, sensitize cancer cells to conventional chemotherapy without undesirable toxic effects. They also do not show their pharmacological activity [47].

It has been noticed that inhibition some signal pathways, for example PI3K/AKT pathway, can ability to reverse cancer MDR. Zhang et al. have been reported that, through the specific inhibition of PI3K 110α and 110β subunits by BAY-1082439, which is a highly selective PI3K inhibitor and via Crispr/Cas9 gene knockout method, P-gp/ABCB1 and BCRP/ABCG2 were downregulated and the drug sensitivity was reestablished in human epidermoid carcinoma and non-small cell lung cancer (NSCLC) MDR cells. [48].

Another method of abolishing MDR related to membrane transport activity is the use of antibodies against this transporter [37] and small-molecule kinase inhibitors—sunitinib or apatinib. They can inhibit the transport proteins of the ABC family by blocking their active sites responsible for ATP hydrolysis and providing the energy necessary for the ejection of substrate molecules from the cell [41].

#### Modulators of Natural Origin

**Figure 1:**
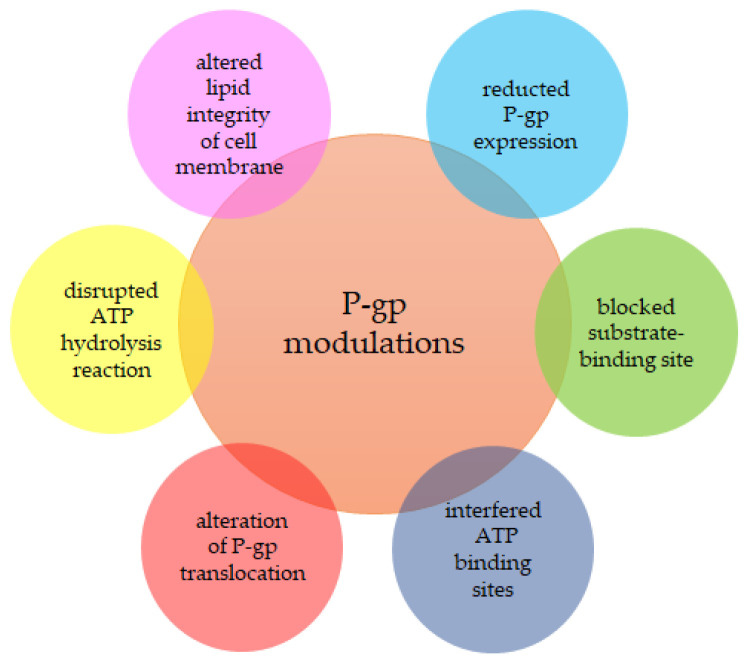
Mechanisms of P-gp modulations [31,34,48,49,50,51,52,53].

A large proportion of the flavonoids are specific inhibitors of the P-gp transporter [31,49]. Several mechanisms can modulate P-gp. The substrate-binding site may be blocked [34], the ATP binding sites may be affected [50], the ATP hydrolysis reaction may be disrupted [51], the lipid integrity of the cell membrane may be altered [52], and the level of P-gp expression can be reduced [48,53] (Figure 1).

Compounds that inhibit ATP hydrolysis are potentially better inhibitors of P-gp because they are unlikely to be P-gp substrates. As a result, such substances can be applied at a low dose. So far, no modulator has been found that can interact with the ATP binding site to disrupt the P-gp ATPase catalytic cycle. Substances with such a mechanism of action are sought. They would provide better inhibitors with high specific activity. The modulation mechanism induced by plant modulators is largely undefined [31]. It was shown that some flavonoids could change the order of lipid packing in the membrane and thus change the fluidity or permeability of the membrane [54]. The influence of flavonoids on drug accumulation may result from their non-specific interaction with the cell membrane, which results in the increased passive permeability of the membranes [55]. Another possibility may be a flavonoid-induced decrease in P-gp expression in P-gp overexpressing cells. One of the potential mechanisms responsible for modulating the action of P-gp by flavonoids may also be the inhibition of P-gp ATPase through the direct interaction of the flavonoid with the ATP binding site [56]. It has been shown that flavonoids, including genistein, catechin gallate, epigallocatechin gallate, epicatechin gallate, and silymarin, can directly bind to the P-gp substrate via the substrate’s P-gp binding site [57]. Besides, it was found that some flavonoids directly interact with the ATP-binding domain of P-gp [58]. However, different flavonoids may interact differently with P-gp since opposite effects on P-gp ATPase activity were observed for various flavonoids [59]. The structural activity of the flavonoids has a significant influence on the activity of the P-gp interaction. The presence of the 5-hydroxyl group, the 3-hydroxyl group, and the double bond causes a strong interaction of the flavonoid with the ATP-binding domain in P-gp [59]. Various results were reported regarding the P-gp modulating activity of some flavonoids. Yeh et al. demonstrated that kaempferol and quercetin stimulate doxorubicin efflux mediated by P-gp [60,61], while Scambia et al. reported that quercetin inhibits doxorubicin efflux from tumor cells [62]. Shapiro and Ling showed that quercetin inhibited P-gp-mediated transport of the fluorescent probe, at least in part by inhibiting P-gp ATPase activity [63]. The efficacy of chronic oral chemotherapy is highly dependent on the P-gp transporters present in the intestinal endothelium. Flavonoids can be nontoxic P-gp inhibitors, significantly improving the bioavailability of chemotherapeutic agents. Choi et al. conducted numerous studies based on the inhibition of P-gp with flavonoids. In vivo studies showed that orally administered paclitaxel and flavone (naringin) significantly increased paclitaxel bioavailability compared to samples where paclitaxel was used alone [64]. First-generation P-gp modulators have undesirable side effects, which explains the interest in flavonoids as nontoxic P-gp inhibitors. The importance of flavonoid-transporter interactions in the pharmacokinetic mechanisms is yet to be well demonstrated. Therefore, further detailed studies are needed to assess the in vivo impact of flavonoids on the bioavailability of drugs and identify and predict potential interactions with drugs [31].

In the past decade, knowledge of weak points of MDR mechanisms enabled scientists to develop new strategies against MDR cancer cells. In addition to the above-mentioned MDR modulators, a lot of novel potential anticancer agents have been designed to overcome these mechanisms [65,66].

## 3. MicroRNAs as Regulators of MDR Gene Expression

As was described earlier, one of the major problems of clinical treatment in anticancer therapy is multidrug resistance (MDR). The MDR mechanism includes active drug efflux transport of ABC superfamily of proteins, which destroys chemotherapeutic effectiveness and undoes/defeats anticancer therapy. The goal of researchers is to find a way to outsmart these mechanisms. Next to the strategies to overcome MDR in cancer cells described above, others that involve the use of microRNAs (miRs or miRNAs) are being developed.

The so-called miRs represent a class of single-stranded non-coding RNAs with a length of approximately 20 to 25 nucleotides [67]. They have significant roles in the post-transcriptional control of gene expression. They act through RNA interference by forming imperfect hybrids with target mRNAs leading to mRNA degradation or translation inhibition [67] by binding to the target mRNA 3′-UTR, 5′UTR, or the coding region [68,69]. In rare circumstances, dependent on cell cycle and co-factors, miRs can also function as positive regulators of gene expression by binding to protein-coding exons or the 5′UTR region. It was suggested that miRs might also bind to the promoter region of genes [70,71,72,73].

Significant alterations of miR expression profiles were observed between cancerous cells and normal tissues from the same organ or in drug-resistant cancer cells compared to parental drug-sensitive cancer cells. This finding gives rise to the search for miRs that could regulate MDR.

P-gp is the best-characterized efflux pump mediating MDR. Its regulation appears to be a complex and highly controlled process that can be predominantly regulated at the transcriptional and post-transcriptional levels. The miRs reported as MDR-1/P-gp regulators and reviewed in [74] miRs described as direct regulators of P-gp cause an inverse correlation between the expression of miRs and P-gp mRNA levels. The decrease in MDR-1 expression and consequently an increase in cellular sensitivity to doxorubicin was observed after the increase of the cellular levels of miR-451 in a doxorubicin-resistant breast cancer cell line (MCF-7/DOX) [75], miR-331-5p in resistant K562/DOX leukemia cells [76], and miR-27a in a chronic myeloid leukemia cell line (K562) [76]. This group of direct regulators also includes miR-298, which was significantly downregulated in the doxorubicin-resistant MDA-MB-231 cells compared to the doxorubicin-sensitive MDA-MB-231 cells [77]. It was reported that miR-298 downregulation caused an increase of P-gp expression and induced resistance to doxorubicin in breast cancer cells (MDA-MB-231) [77]. Direct regulation of MDR-1 was also described for miR-145. The downregulation of miR-145 in Caco-2 cells increased P-gp expression but not MDR1 mRNA level [78]. Feng et al. showed that miR-331–5p and miR-27a are inversely correlated with the expression of a drug-resistant factor, P-glycoprotein (P-gp), in leukemia cell lines with gradually increasing resistance [76].

Moreover, miRs can also act at the post-transcriptional level as indirect regulators by targeting other mRNAs that code for intermediate proteins or transcription factors engaged in MDR-1 gene activation and affecting other factors, which modulate P-gp expression. As mentioned above, direct regulator miR-27a can also indirectly modulate MDR1 mRNA and P-glycoprotein expression by targeting homeodomain-interacting protein kinase-2 in human ovarian cancer cells [79]. It was shown that miR-508-5p could associate with the 3′- UTR regions of both ABCB1 and zinc ribbon domain-containing 1 (ZNRD1) and suppress their expression at mRNA and protein level [80]. As a result of overexpression of miR-508-5p in human gastric cancer cell line SGC7901, the resistance to multiple chemotherapeutics in vitro and tumor sensitivity to chemotherapy in vivo were reversed [80]. By increasing the level of miR-21 via the presence of hyaluronan, a decrease in the levels of the tumor suppressor protein programmed cell death 4 (PDCD4) was observed in a doxorubicin-resistant breast tumor cell line MCF-7. Using an anti-miR-21 inhibitor to silence miR-21 expression enhances the PDCD4 expression [81].

The indirect upregulating impact of the miR on P-gp expression via repressive transcription factors, such as BMI1 polycomb ring finger oncogene (BMI-1), cyclooxygenase 2 9COX20, homeodomain-interacting protein kinase 2 (HIPK2), macrophage migration inhibitory factor (MIF), Y-box binding protein 1 (YB1), zinc finger E-box-binding homeobox 1 (ZEB1), was reviewed in [82]. The increase in the cellular level of some miR was shown to increase the sensitivity of resistant cells to cisplatin, epirubicin, paclitaxel, doxorubicin, vincristine, and fluorouracil [73]. In addition to post-transcriptional regulation, the involvement of miR-27a acting at the transcriptional level was reported. The downregulation of miR-27a could reverse drug resistance and decrease the expression of P-gp in ECA-109 squamous cell carcinoma of the esophagus [73] and MKN45 gastric cancer cells [83]. Zhao et al. [71] postulated that the upregulation of miR-138 could downregulate P-glycoprotein expression and the transcription of the MDR-1 gene. Additionally, overexpression of miR-138 promotes adriamycin-induced apoptosis in leukemia cells [83].

Other recent works also demonstrated that some miRNAs might regulate the expression of the next ABC through their actions on ABC 3′UTR or miR-210-3p negatively regulated ABCC1 and improved drug-sensitivity of renal cell carcinoma Caki-2/VBL and Caki-2/DOX [84]. Overexpression of miR-328 in MCF-7/MX100 breast cancer cells decreased the level of ABCG2 mRNA and protein and, more importantly, increased sensitivity to mitoxantrone [85]. The researchers cannot exclude the possibility that miR-328 also targets the transcriptional factors of ABCG2, leading to indirect transcriptional regulation of ABCG2 [85].

It was shown that an increased miR-200c level might cause an indirect transcriptional regulation of ABCG2 by targeting transcription factors such as BMI-1 in melanoma cells [86] and ZEB1 in human breast cancer cells [87].

A search for potential regulatory miRs may occur with the participation of bioinformatics tools. Medarova et al. [88] chose unexplored miRs with high nucleotide sequence correspondence to two representative MDR proteins, MGMT and ABCB1. They demonstrated that therapeutic miRs could be identified based on the nucleotide sequence matching miRs to targeted mRNA.

A diversity of miR expression profiles and the observed significant influence of miR level changes on MDR gene expression modifications indicate that miRs could be ideal biomarkers of diseases and candidates in drug therapy management.

## 4. Anticancer Therapies Targeting Ion Channels

As part of cell membranes, ion channels (IC) play an important role in ion homeostasis, cellular osmolarity, and signaling regulation, contributing to the cell cycle, proliferation, migration, adhesion metabolism, and apoptosis. The extra- and intracellular environment changes during neoplastic transformation induce remodeling of the cell membrane and modifications in ion channel expression and activity. The aberrant expression of a wide range of ion channels was detected in many types of cancers as reviewed elsewhere [89,90]. Inversely, altered expression and activity IC can lead to tumorigenicity and the formation of metastatic potential. These findings stimulate research into the practical implementation of ion channels as targets in cancer diagnostics and therapy. This chapter focuses on the main anticancer strategies based on IC and discusses examples of their application. As a complementary review of ion channel inhibitors, we refer to [91].

The regulation of IC is a key element in the treatment of many cardiovascular and neurological diseases. Natural and synthetic inhibitors of ion channel activity used in such therapies are potential anticancer drugs. Imipramine, used as an antidepressant drug, inhibits potassium voltage-gated channel KCNH1. Interestingly, KCNH1 is reported to be upregulated in many cancers, including breast, lung, prostate, and colon; thus, its use is postulated as an early tumor marker and prognostic marker as well [92]. It has recently been demonstrated that imipramine can be successfully adapted to suppress DU145 prostate cancer cells [93]. However, this tricyclic antidepressant also interacts with other ion channels on neuronal and cardiac cells, which may lead to different cardiovascular side effects; thus, its clinical application may be limited [94,95]. On the other hand, imipramine blue, its analog, inhibits progression and metastasis of breast cancer cells, although the compound’s low toxicity was proven only in the animal model [96]. Another member of the potassium voltage-gated channel is KCNH2 which is inhibited by fluoxetine (Prozac), a popular serotonin reuptake inhibitor and a neurological disorder drug. Interestingly, fluoxetine also enters mitochondria, where it inhibits ATP production. Consequently, Ca^2+^ leakage from the endoplasmic reticulum is accompanied by ORAI-1- mediated extracellular Ca^2+^ entry, leading to an increase of ion concentration in the cytoplasm and mitochondria [97]. The anticancer properties of fluoxetine were confirmed in many cancer models, both in vitro and in vivo [98,99]. However, some recent studies support its pro-survival potential as well [100,101]. An example of antiarrhythmic agents that can be potentially used in anticancer therapy is verapamil. It acts mainly as an inhibitor of L-type calcium channels, although it was reported to block KCNH2 [102,103]. Antiproliferative verapamil activity was shown in many neoplasms, among others in melanoma and breast cancer [104,105]. Moreover, verapamil enhances paclitaxel’s effectiveness in chemoresistant cancer cells via ABCB1 (MDR1) modulation [106]. Similarly, many other antagonists of IC are used in combination with well-known anticancer drugs with satisfactory results. Mibefradil, an antihypertensive drug, blocks T-type calcium channels and KCNH1, giving promising results together with temozolomide in high-grade glioblastoma therapy. It is noteworthy that despite profitable inhibition of Ca^2+^ influx into tumor cells by mibefradil, it should be used with the utmost care due to the risk of cross-reactions with other drugs [107].

Many natural compounds can regulate the activity and expression of ion channels. Calcitriol is an endogenously synthesized compound, which modulates intracellular signaling pathways through the vitamin D receptor (VDR). Among others, the KCNH1 expression is decreased in calcitriol- treated cancer cells. Interestingly, therapy combined with astemizole enhances calcitriol bioavailability and reduces its dose, leading to growth inhibition of breast cancer cells in vitro and in vivo [108,109]. Moreover, concomitant administration of calcitriol with other natural compound resveratrol or curcumin results in reduced tumor volume of triple-negative breast cancer cells [110]. Furthermore, resveratrol can induce the expression of ATPase sarcoplasmic/endoplasmic reticulum Ca^2+^ transporting 3 (ATP2A3, SERCA3) in breast cancer cells. As a consequence, the concentration of cytoplasmic calcium ions changes, which stimulates death. It has been recently shown that resveratrol induces SERCA3 through epigenetic modification of the *ATP2A3* gene [111,112]. In contrast, curcumin and its derivative RL71 (3,5-bis(3,4,5-trimethoxybenzylidene)- 1-methylpiperidin-4-one) directly inhibit ATP2A2 (SERCA2) and activate ER-stress associated apoptosis [113].

It was demonstrated that some natural animal toxins interact directly with voltage-dependent potassium, chloride, and calcium channels. Chlorotoxin (CTX) is a neurotoxic peptide originally found in the venom of an Israeli scorpion, which inhibits chloride channels. Some evidence indicates that the main target of CTX is metalloproteinase 2 (MMP-2), which can form a complex with chloride voltage-gated channel 3 (CLCN3). The endocytosis of the complexes induced via binding of CTX contributes to chloride ion efflux in cells [114,115]. Synthetic chlorotoxin derivatives were used successfully in both preclinical and clinical trials of glioma therapies [116,117,118]. Potassium transporters play a crucial role in cell membrane potential. Concomitantly, the deregulation of the K^+^ channel expression is predominantly observed in many tumors [119]. Iberiotoxin (IBTX) and charybdotoxin (CTX) are other scorpion venom peptides, selective modulating a voltage-dependent large-conductance Ca^2+^ activated K^+^ channel (KCNMA1). Human metastatic breast cancer cells with overexpression of KCNMA1 have diminished the migratory and invasive properties after treatment with IBTX. Similar effects were observed in cells with direct silencing of KCNMA1 expression through RNAi [120]. Furthermore, IBTX turned out to be an effective inhibitor of prostate cell proliferation, while CTX decreased the migration potential of melanoma cells [121,122]. Potassium small conductance calcium-activated channels are inhibited by other scorpion toxins of the αKTx5 family (e.g., apamin, scyllatoxin, PO5), which differ in the channel member selectivity. Among them, tamapin exhibits the highest selectivity for the KCNN2 channel and induces the death of Jurkat T cells and MDA-MB-2 breast cancer cells [123,124]. Recently, the mutant E25K/K27E of tamapin has been shown to be an effective blocker of KCNN3, leading to inhibition of MDA-MB-435s cell migration. These results indicate that directed-mutagenesis of known, natural ion channel inhibitors can be used in drug design for selective anticancer therapies [125].

Hypothetically, good selectivity of IC inactivation and anticancer treatment should lead to immunotherapies based on monoclonal and polyclonal antibodies. However, this strategy is mainly applied for sensitive and selective cancer cell detection, although some use whole antibodies or their fragments directly in cancer therapies has been presented [126]. The application of polyclonal antibodies, targeting 17-amino acid fragments of non-functional ATP-gated receptor P2X_7_, confirmed its therapeutic potential in Phase I of the clinical trial concerning basal cell carcinoma [127]. P2X_7_ is a membrane receptor working as a non-selective cation channel in response to rapid exposure of ATP. However, prolonged ATP stimulation causes uncontrolled pore opening and allows the transport of larger molecules that regulate the survival/death balance in the cells. Simultaneously, high ATP concentration provides the dominant expression of the non-pore functional form of P2X_7_ (nfP2X_7_), essential for cancer cell survival, which indicates that nfP2X_7_ can be a selective and attractive target for cancer therapies. [128]. Despite the remarkable development of many anticancer therapies based on the chimeric antigen receptor T-cells, there are no studies on targeting ion channels by this method to our knowledge.

Alternative methods directed at regulating ion channel expression in cancer cells could include gene therapies based on RNAi or CRISPR/Cas9. This strategy allows for satisfactory results with no effects on other ion channels compared to most chemical blockers. Transient receptor potential melastatin 2 (TRPM2) belongs to non-selective cation channels. Its role in regulating cancer growth and metastasis was confirmed by many studies [129,130]. On the other hand, selective downregulation of TRPM2 via shRNA results in the proliferation decrease and apoptosis promotion in gastric and lung cancer cells. Notably, TRPM2 silencing also enhances the effectiveness of chemotherapy with paclitaxel and doxorubicin [131,132]. Similarly, the use of selective shRNA and siRNA targeting KCNH1 and ORAI1 leads to an increase in cancer cell sensitivity to chemotherapeutic drugs [133,134]. Furthermore, possible microRNA- and CRISP/Cas9-mediated IC expression was also found [135,136]. However, these strategies are usually limited to cellular models, probably due to ethical and legal restrictions.

Electromagnetic field or photothermal therapy stimulates cellular internal and external membranes, leading to their remodeling. One of the consequences of using such methods is the regulation of multiple ion channels simultaneously, which leads to altered ion fluxes. Despite poor selectivity, both electromagnetic field pulses and photodynamic reactions are shown to be efficient in many anticancer therapies [137,138,139]. On the other hand, the exact mechanism of these stimuli is still poorly understood and difficult for prediction due to the multifactorial nature of induced processes [140].

## 5. Plasma Membrane Receptors and Modulations

### 5.1. The Mechanism of Signal Transduction from The External Environment to The Cell by Integral Membrane Receptor

Normal and pathological eukaryotic cells are surrounded by a cell membrane that protects them against damaging factors and allows them to contact the external environment [141]. Eukaryotic cells have the capacity to receive, select, analyze and respond to signals received from the environment and other cells of a multicellular organism. Therefore, intercellular signaling is a necessary process for the functional integration of multicellular organisms. Many aspects of cell function, such as metabolic regulation, survival, proliferation, differentiation, and death, are dependent on appropriately effective systems involved in cell signaling. Chemical compounds, called ligands, transmit information and play a significant role in balancing and synchronizing life processes throughout the cells and the body [141]. Communication between single cells in a multicellular organism acts by synthesizing ligands, either released or presented on their surface, that interact with receptors, either inside the target cell or on its surface. Ligands are released in small, usually volatile or soluble molecules, including proteins, peptides, fatty acids, steroids, gases, and other low-molecular-weight compounds [141].

**Figure 2:**
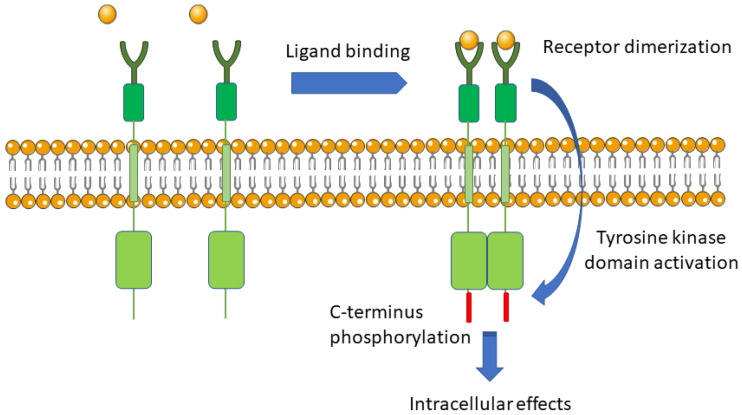
The schematic mechanism of signal transduction via the membrane receptor [141].

The membrane receptors associated with signaling are integral membrane proteins. Their structure includes extracellular (acceptor), transmembrane, and intracellular (effector) parts. The acceptor domain binds ligand specifically and reversibly. The transmembrane part anchors the receptor in the cell membrane. The effector domain can generate and amplify the signal inside the cell (Figure 2).

**Figure 3:**
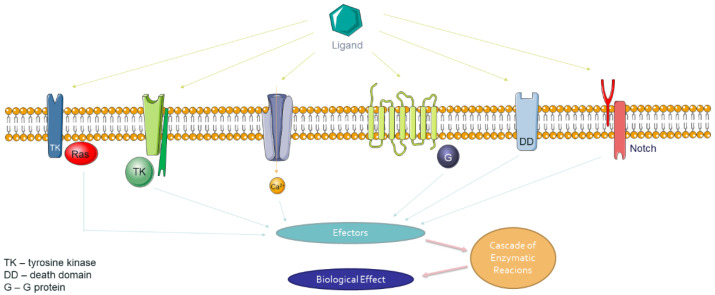
The variety of the signal transduction mechanisms via different membrane receptors [142,143,144,145].

Based on the number and nature of transmembrane domains, three classes of membrane receptors are distinguished [141]. Class I contains receptors comprised of several protein subunits, each repeatedly pierces the cell membrane, that together form an ion channel, such as ionotropic receptors (IRs) [142]. Class II consists of receptors composed of one peptide chain, which penetrates the cell membrane several times; for example, G protein-coupled receptor (GPCR) penetrates the membrane seven times [143]. Class III includes receptors that penetrate the cell membrane once. The latter may have a monomeric (receptors of most growth factors) or oligomeric structure (insulin and most cytokine receptors). Class III receptors have the amino acid sequence with enzymatic activity (e.g., non-receptor tyrosine kinases [144] or contain the so-called death domain (DD) [145] (Figure 3)).

Ligand-activated receptors transmit signals into the cell by activating signaling pathways that ultimately affect cytosolic machinery or nuclear transcriptional programs or directly translocating the nucleus to regulate transcription. The abundance of chemical signals forces the cells to recognize and correctly interpret them by much more diverse information carriers. The proper cellular response selectivity toward the stimulus and specificity of the achieved response is regulated by receptor proteins that can bind to excitation signal-bearing molecules, and at the same time, transmit this excitation deep into the cell, up to executive systems called effectors [141].

### 5.2. Reaction Required and Amplification Mechanism

One of the key mechanisms that control signal transmission by receptors is exchanging the information, referred to as cross−talk. There are different types of interaction between intracellular signaling pathways: phosphorylation of receptors and regulatory proteins by kinases [146], the interaction of intracellular signal transduction pathways [147], effects on intracellular calcium release [148], heterooligomerization of various receptors of the same family of ligands [149]. For example, the signaling via kinase-associated receptors is organized into multiple feedback mechanisms to confirm an appropriate transmitter level. Therefore, many tyrosine kinases and cytokine receptors bind protein tyrosine phosphatases (PTPs), including SHP1 and SHP2, that contain Src-homology 2 (SH2) domains [150]. Thus phosphorylation/dephosphorylation cycles PTPs modulate signaling quantitatively as well as qualitatively. Another example of cross−talk is the heterotrimeric G protein cascades Rho guanine nucleotide exchange factors (RhoGEFs), which can serve as the direct downstream effectors of heterotrimeric G proteins [151]. It was postulated that RAS signaling is essentially involved in the switch from tumor-suppressive to tumor-promoting functions of the TGF-β family, leading to enhanced cancer growth and metastatic dissemination of primary tumors [152]. Extensive cross−talk between the different pathways activated upon platelet-derived growth factor (PDGF) stimulation was observed. Mendelson et al. noted triggering a metalloproteinase-dependent cross−talk between the PDGFRβ and the epidermal growth factor receptor (EGFR)/ERK1/2 signaling axis and indicated the involvement of metalloproteinase ADAM17 upon activation of the PDGFRβ [153].

### 5.3. Participation of Integral Membrane Receptors in the Pathological Metabolic Pathway and Targeted Therapy

Membrane receptors are responsible for the precise reading and transmission of information from ligands to effectors, which activate metabolic pathways. Each modulation of receptors, e.g., genetic defect, may change information transfer and activate different metabolic pathways. Much evidence indicates that receptor dysfunction leads to various diseases, mainly tumors [154]. The observation that overexpression of many integral membrane receptors is common in different cancers became the basis for developing new anticancer therapies based on the inhibition of membrane receptors. Numerous antagonists were developed, including inhibitory antibodies, ligand traps, and low-molecular-weight kinase inhibitors, for use in targeted therapies, which is very promising in treating cancer without side effects [155,156]. Many of these antagonists are now being applied in clinical treatment or undergoing clinical trials. The serine/threonine kinase and tyrosine kinase receptors often transmit growth inhibition and apoptotic pathways and have tumor-suppressive effects [157,158,159,160].

Due to the fact that overactivity of receptor tyrosine kinases (RTKs) is related to developing various malignancies, we focus on their dysfunctions, ways of modulation, and potential application in targeted therapies. RTKs have a high affinity for many polypeptide growth factors, cytokines, and hormones. Modulation or dysregulation of this receptor leads to developing numerous diseases, including cancer and others, characterized by excessive proliferation of cells, inflammatory and fibrotic conditions [157,158,159,160].

Platelet-derived growth factor receptors (PDGFRs) belong to a subfamily of human RTKs. Different dimeric isoforms of the ligand induce the formation of different dimeric complexes of PDGFα and PDGFβ receptors. Point mutations in Kit and Alpha (PDGFRa) were found in gastrointestinal stromal tumors (GISTs). Moreover, patients with familial GIST syndrome have constitutional KIT/PDGFRA mutations [161]. The translocated kinase domains of PDGFRs and fibroblast growth factor receptors (FGFRs) can act in the cytoplasm as fusion proteins with constitutive activity characteristic of several rare leukemias [162]. Amplification of the ERBB2 gene was found in 20% of breast cancer cases and a mutated version of the epidermal growth factor receptor (EGFR) gene in 30% of glioblastomas [119,163].

The development of targeted therapies has recently become a crucial research goal. Many small-molecule inhibitors such as lapatinib, neratinib, and tucatinib were used for treating cancer caused by the mutations in RTKs [164]. These inhibitors precisely join the ATP-binding pocket of the intracellular tyrosine kinase domain [155]. Another strategy involves using monoclonal antibodies that inhibit RTK activation. This is exemplified by applying cetuximab in lung cancer, panitumumab in colon cancer, cetuximab in head and neck cancer, or trastuzumab and pertuzumab in breast cancer [165,166,167,168].

Some currently applied strategies specifically disturb oncologically active cells by targeting surface receptors and endogenous signaling molecules. The dysfunction of the ErbB2 (HER2) receptor plays a key role in breast cancer pathogenesis. For this reason, HER2 is an important molecular target for researchers. Some antibodies directed against the growth factor receptors HER2 and EGFr were approached [163]. Although HER2+ breast cancers grow quicker and are more invasive than other types, thanks to applying the antibodies directed against HER2 receptors, they are generally approachable to anti-HER2 therapy, which significantly enhances the prognosis [163,169].

## 6. Modulation of Cell Membrane Lipidomics

Lipidomics is a complete set of lipid variants and their biological functions in relation to the expression of proteins responsible for lipid metabolism and their functions. Membrane lipids are essential elements of the cell membrane that determine many cytophysiological processes [170]. The growing understanding of the lipid structure of biological membranes and changes in their properties in various diseases initiated the development of therapies aimed at membrane modification. Therapeutic strategies may involve direct interference with the composition of membrane lipids, which determines the activity of transmembrane proteins and modifies the organization of membrane microdomains, which are involved in transmitting cellular signals [3]. They may also concern changes in the activity of membrane-related enzymes or modulation of gene expression of enzymes influencing lipid composition. Under the influence of phospholipid composition changes and the length and number of double bonds of esterified fatty acid acyls, lipids in the membranes temporarily change their order. There is a liquid-disordered (Ld) phase, where lipid packing is the loosest, and lateral diffusion and lipid rotation are frequent, and a liquid-ordered (Lo) phase, where the lateral lipid diffusion is maintained, but rotation around the long axis of the alkyl chain is significantly limited [171,172]. Such an organization of lipids occurs in membrane lipid microdomains, called lipid rafts, high in cholesterol [173].

The lipid profile analysis of normal and neoplastic cell membranes shows significant differences in the lipid composition. Breast cancer cells have higher cholesterol content compared to normal cells [174]. Neoplastic cell membranes also show disturbances in the ratio of phosphatidylethanolamine (PE) to sphingomyelin (SM) [175]. Lipid mobility is important in the lipid-protein interaction, which is largely dependent on the cholesterol and sphingomyelin content in the raft microdomains [2]. Studies conducted on artificial membranes indicated that daunorubicin by binding to phospholipids reduces the microdomains with hexagonal lipid organization, which disrupts the lipid-membrane protein interaction, and ultimately modifies cell signaling and vesicular transport [176]. Furthermore, 2-hydroxyoleic acid effectively reduces the lipid order in the membrane by stimulating sphingomyelin synthase and increasing the content of sphingomyelin in the membrane of lung cancer, leukemia, and glioma cells, while the lipid structure of normal cells remains unchanged [175].

Barceló-Coblijn et al. indicated that with an increase of the SM:PE ratio, the MAPK kinase pathway is inactivated in tumor cells [177]. Changes in the lipid phase in membranes can be achieved using derivatives of polyunsaturated fatty acids (PUFA). It was shown that increasing the amount of unsaturated fatty acids reduces the Lo/Ld phase ratio by almost 10% and reorganizes lipid microdomains [178,179]. Siddiqui et al. demonstrated that the combination of propofol with PUFA reduces migration and adhesion of breast cancer cells and increases cell apoptosis by about 40% [180]. One of the approaches in anticancer therapy is to increase the Ld phase of the membrane. It is possible by applying molecules such as alkylphosphocholine (APC), in which the glycerol backbone was removed [181,182]. Such kinds of molecules include miltefosine, perifosine, and euphrosine [183,184]. Van der Luit et al. demonstrated that SM is necessary for APC binding in rafts, and among alkylphosphocholines, edelfosine most strongly inhibits phospholipid synthesis and induces apoptosis in leukemic cells. The mechanism of APC derivatives is most likely based on the inhibition of phosphatidylcholine biosynthesis in the ER [185]. As a result of increasing the number of ceramides in the membrane, the disruption of calcium ions homeostasis occurs, followed by activation of apoptosis and inhibition of the Ras/Raf/MAPK/ERK PI3K/Akt proliferative signaling pathways [186].

Modifications of membrane lipids may include direct interference with the lipid composition, which determines transmembrane protein activity. They may also include changes in the activity of enzymes associated with membranes or modulating gene expression of enzymes affecting lipid composition. The membrane modifications lead to a change in the lipid-protein and protein-protein interactions; thus, the signaling pathways determining tumorigenesis can be inhibited [183,187].

## 7. Electroporation

Short high-voltage electric pulses modify lipid bilayer structure and induce the creation of temporal aqueous pores in a cell membrane. The technique widely known as electroporation (or pulses electric field—PEF) increases cell membrane permeability and allows for transmembrane transport of different molecules. It is used in many fields such as medicine, biotechnology, or food technology. The size and number of pores depend on the electric pulse parameters (duration, amplitude, shape, number of pulses, etc.) [188]. After application of PEFs, the changes induced depend on the intensity of the applied electric fields and can be either reversible (cells recover their integrity) or irreversible (IRE) when the cells turn necrotic. In accordance with the duration of pulses, the three main types of electroporation are discerned, i.e., nanosecond (nsPEF), microsecond (µsPEF), and milisecond electroporation (msPEF) [189]. The range of electric field generated during the microsecond and millisecond PEF is between 0.1 to 2 kV/cm, while nanosecond PEF uses electric pulses strength above 10 kV/cm. In this chapter, we focus on different modifications of outer and inner cell membranes caused by PEF.

### 7.1. The Aqueous Pore Formation

In natural physiological conditions, eukaryotic cells maintain resting transmembrane voltage (TMV) of −40 to −70 mV between the inner and outer sides of the membrane. The composition of pumps and ion channels is responsible for TMV regulation. An exposition of cells to external electric pulses generates induced-TMV, which may dramatically exceed the ranges of resting TMV and induce structural changes in molecule composition. The type of PEF determines the size and number of pores—nsPEF creates approximately three times more pores compared to the micro- and millisecond), but simultaneously pore diameters are smaller in case of exposure to nsPEF than to longer pulses [190,191]. The research showed that microsecond electroporation affected TMV of the plasma membrane and endoplasmic reticulum membrane, while application of nsPEF impacted plasma membrane, nuclear membrane, endoplasmic reticulum membrane, inner and outer mitochondrial membrane [190]. Molecular modeling, as well as empirical studies, are consistent with TMV-mediated aqueous pore creation. As a result of PEF application to both sides of the phospholipid bilayer, the so-called water fingers or water defects are formed, grown across the hydrophobic width, and the hydrophilic groups of phospholipid bilayer are directed toward water molecules. Pores extend and fill with water allowing the entrance of other ions and molecules such as genes or drugs [189,192].

### 7.2. Effects of The Electric Field on Oxidative Stress

Many years ago, it was noticed that the properties of cell membranes and lipid bilayers could be altered by exposure to electric pulses due to oxidation of their lipid constituents [193,194,195]. Oxidative stress directly impacts the physical properties of cell membranes and therefore plays a vital role in the electroporation process [196]. The imbalance between the generation and the removal of reactive oxygen species in the body is known as oxidative stress. Reactive oxygen species (ROS) are chemical compounds that can participate in chemical reactions, containing oxygen atoms with an unpaired electron or bonds between two oxygen atoms in their structure. Oxygen is several times more soluble in organic solvents than in water. ROS can arise as a result of ionizing radiation, ultraviolet radiation, ultrasounds, photosensitizers, xenobiotics. The most reactive but short-lived oxygen species include hydroxyl radical, hydroperoxide radical, and superoxide anion radical. Reactive oxygen species with bonds between two oxygen atoms include singlet oxygen, hydrogen peroxide, and ozone [197,198,199,200]. The imbalance in the removal of reactive oxygen species by the body due to various pathogens or external factors causes an increase in free radicals. ROS cause damage to cell organelles: the cell membrane, mitochondria, Golgi apparatus, endoplasmic reticulum, lysosomes, and the cell nucleus [197]. Free radicals target proteins (aggregation, oxidation, phosphorylation, dephosphorylation, denaturation), lipids (peroxidation), carbohydrates, and nucleic acids. Changes in the structure of these compounds lead to mutations and cytotoxic effects that affect cell dysfunction [197,201]. The relationship between the use of nanosecond pulses [202,203] and conventional electroporation [204] in the presence of excess reactive oxygen species and initiation of apoptosis may lead to an improvement in the efficiency of electroporation and electropermeabilization procedures [205]. The use of millisecond pulses during electropermeabilization produces reactive oxygen species. The increase in oxidative stress was observed when reversible electropermeabilization was applied. Irreversible electropermeabilization disrupted cell membranes, which inhibited the increase in oxidative stress. On the other hand, applying a series of short microsecond pulses caused a decrease in cell survival, which was not related to an increase in oxidative stress [195,206].

### 7.3. Effects of the Electric Field on Lipid Peroxidation

Lipid peroxidation (LPO) is a free radical oxidation process of unsaturated fatty acids or lipids from which the peroxides of these compounds are formed [197,207,208]. There are three stages of the lipid peroxidation process. The first step consists of detaching the hydrogen atom from the unsaturated fatty acid molecule, which is part of phospholipids that are easily oxidized by free radicals [197]. LPO can be initiated by hydroxyl, peroxide, alkoxy, or alkyl radical, as well as ozone, nitrogen oxide, and dioxide, or sulfur dioxide and hypochlorite [209]. In the second step, free alkyl radicals react with oxygen to form peroxide free radicals. In turn, these radicals can detach hydrogen atoms from successive unsaturated fatty acids, which in turn leads to further reactions with unsaturated fatty acid molecules. This step is repeated many times. In the third step, reactions occur between two free alkyl or peroxide radicals or two different ones nearby [207].

The exposure of cells to electrical pulses can cause LPO, which was confirmed in plant cells [195,210], bacteria [211,212], and mammalian cells [193,195,213]. LPO induced through electroporation causes growth of membrane permeability and fluidity [195]. Studies including micro- and millisecond pulses demonstrated that electric pulses provoke the generation of reactive oxygen species (ROS) and intense oxidative damage of unsaturated lipids in cell membranes. Free radicals formed during lipid oxidation can react with other components of cell membranes, e.g., proteins. The end products of LPO may be aldehydes, as was confirmed by measuring the concentration of malondialdehyde (MDA) [193], hydroxy−aldehydes [197,208], and hydrogen peroxide [214,215] by detection of superoxide anion radicals [189,194].

Many studies show that the use of appropriate electroporation protocols induces the formation of lipid hydroperoxides in cell membranes [207,216]. It was observed that electrical pulses generate reactive oxygen species and ROS concentration and increase with electric field intensity [193,194,195,213,217], pulse duration, and the number of pulses [194]. It was found that there is a correlation with cell membrane permeability [194,195,213], membrane resealing time [194], and cell damage [189,193,194]. Studies show that a controlled increase of LPO in cell membranes prior to electroporation increases membrane permeability without significantly affecting cell survival, which may increase the efficiency of electropermeabilization [205].

### 7.4. Effects of Electric Pulses on Membrane Proteins

There is a hypothesis of denaturation of transmembrane transport proteins during exposure to electric pulses. Electropermeabilization can generate sufficient heating membrane proteins for denaturation. Cell membrane takes up to ten minutes to excise or replace denatured proteins from the membrane and regain impermeability [218,219]. The available results confirmed that electroporation of sub-microsecond duration and high amplitudes could affect the conductivity of transmembrane protein structures, i.e., voltage-gated Ca^2+^ and Na^+^ channels [220,221,222,223,224,225,226].

### 7.5. Cytoskeleton Remodeling after PEF

In recent years, the influence of PEF on cytoskeleton structure has been studied extensively. A cytoskeleton is composed of actin, microtubules—tubulin, and intermediate filaments, mainly providing cell stability and cell-cell contact, proliferation, migration, and differentiation [227,228]. In physiological conditions, actin fibers form stress fibers, filopodia, and lamellipodia, determining cell shape, proper tension and flexibility, extra- and intracellular transport, contact between cells, and locomotion. Actin is strictly linked with the cell membrane and interacts with the phospholipid bilayer modification caused by, e.g., PEF. Studies show that electroporation affects ruffled membranes and actin bundles accumulated on the cell surface and periphery [229,230,231]. Changes in cell shape or rounded and swollen cells are often observed after PEF application. The most frequent changes in actin triggered by PEF include shortening, fragmentation, and fiber density loss. Analyses of cytoskeleton disruption also revealed a decline of membrane stiffness and impaired attachment of F-actin fibers to the cell membrane [232]. The electric pulses permeabilize other cell components causing enzyme or/and ions release (e.g., caspase, Ca^2+^), which may lead to cytoskeleton disorganization [233]. While nsPEF causes only membrane ruffling, which disappeared after 4 min (60 ns, 60 kV/cm, 1 impulse). However, longer pulses (100 ns, 20kV/cm, 20 impulses) initiate shortening and oligomerization of actin fibers, and the cytoskeleton recovery is achieved approximately after 60 min [230,234]. The increase of zyxin expression and stress fiber tension was found in normal muscle cells C2C12 after µsPEF application. However, the actin cytoskeleton and zyxin reduction disruption was observed after applying the same PEF parameters [235]. Millisecond PEF (5 ms, 0.4 kV/cm, 8 pulses) causes actin stress fiber disruption even after membrane resealing [236].

Microtubules (MTs) are stiff, tube-like, and localize around the nucleus and radiate to the edge of the cell membrane [237]. PEF application induces a short-term reduction of microtubule network density and depolarization [238,239]. Likewise, µsPEF causes tubulin depolarization and fragmentation, which are recovered after a few hours [240]. Thompson et al. and Carr et al. demonstrated tubulin depolarization and fragmentation after nsPEF. In addition, microtubules accumulated nearby the cell membrane and reduced the network density. Studies indicate numerous ruptures of tubulin filaments [229,241].

Intermediate filament (IF) is the third element of the cytoskeleton and the most insoluble part of the cell components. It creates fibers with high tensile strength and is responsible for stabilizing cells, cell organelles and creating specific junctions. IFs partitioned by their structure are classified into four groups: vimentin filaments, keratin filaments, neurofilaments, and nuclear lamins [236,242]. The studies under PEF interaction with intermediated filaments are insufficient; however, some of them demonstrated perinuclear collapse of IF and disruption of nuclear lamins after nsPEF [238,242,243]. Despite the increasing number of PEF effects on cell structure, many cytoskeleton disruption mechanisms still require an experimental solution.

### 7.6. Clinical Potential (Aspect) of Electroporation

Over the years, electroporation procedures have reached a major role in clinical trials in human and veterinary oncology. Application of electric pulses allows to overcome barrier of cell membrane and transport different molecules into cellular compartments. In oncology, electroporation combined with chemotherapeutics (bleomycin, cisplatin) injection called electrochemotherapy (ECT) results in several facilities: increase of drug cytotoxicity, low drug concentration, significant reduction of tumor size, immune system stimulation and reduction of side effects [244]. Successful ECT clinical results demonstrating effectiveness and safety of the method have become a foundation of European Standard Operating Procedures on Electrochemotherapy (ESOPE) with Cliniporator. Currently, ECT is used against primary tumors and metastasis in over 130 European oncology centers and also in veterinary oncology [245].

Nonthermal irreversible electroporation (NTIRE) uses extremely high electric pulses to cell or tissue ablation. Ones of many advantages of NTIR are: minimal invasive surgical procedure, tissue architecture preservation and scaring reduction [246].

DNA vaccination and gene transfer (GET) are another medical application of electroporation. GET is used in cancer treatment by delivering immunomodulatory (e.g., interleukins) or vasculature tumor targeted genes. There are number of reports of IL-12 and AMEP delivery by GET for treatment of human cutaneous melanoma nodules, horses’ sarcoids, and primary dog tumors [247]. Moreover, GET significantly increases the DNA vaccination effectiveness, while introduction of DNA and RNA into cells is explored in regenerative medicine [248].

## 8. Sonoporation

**Figure 4:**
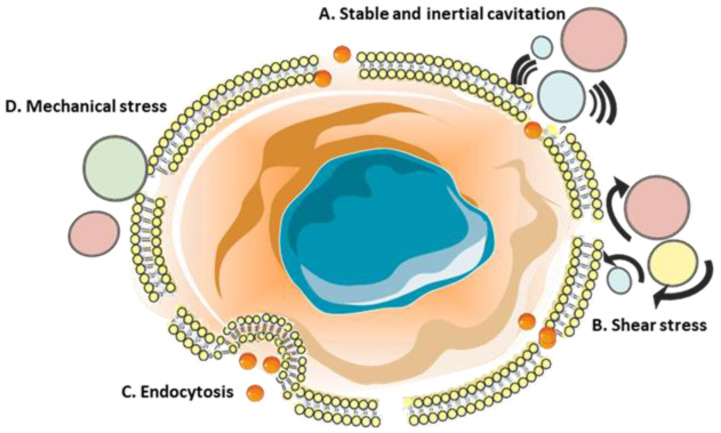
Mechanisms involved in sonoporation of the cell membrane after ultrasounds application: (**A**) cavitation of microbubbles and gas bodies in surrounding medium; (**B**) shear stress evoked by acoustic streaming and medium flow during cavitation; (**C**) endocytosis; (**D**) mechanical stress triggered by the cavitating agents [253].

Over the past few decades, research has revealed the potential to incorporate physical techniques into anticancer therapies to deliver impermeable compounds into the cell interior. One of the most promising is the use of ultrasounds (US) which can stimulate pore formation in the cell membrane structure, referred to as sonoporation. Except for externally delivered ultrasounds, microbubbles are the second component incorporated into this technique. Although not mandatory, they are frequently used together with ultrasounds to increase the sonoporation efficiency through cavitation [249,250,251]. Sonoporation is based on the propagation of ultrasounds through the media and tissues exerting direct radiation and secondary forces resulting from the interaction between the tissue, surrounding media, and microbubbles [252]. Consequently, the main factors contributing to the effectiveness of sonoporation-based therapies include acoustical driving parameters, properties of the environment surrounding the targeted tissue, and additionally introduced cavitation agents [252]. Lentacker et al. identified three main ultrasound settings enabling sonoporation: (i) application of ultrasounds without additional cavitation agents (ii) stable cavitation exerted by low-intensity ultrasound (iii) inertial cavitation evoked by high-intensity ultrasound [253]. The above approaches may provide pore formation through various mechanisms, as shown in Figure 4.

Among the mechanisms triggered by ultrasounds, the most significant role is attributed to the oscillation of cavitation agents (Figure 4A) and shear stress generated by the medium movement around cavitation nuclei (Figure 4B). The gas inside a bubble differs in impedance from surrounding tissues; hence, it shrinks and expands in response to the negative or positive ultrasound phase [253,254,255,256]. The ultrafast coupled microscopy and real-time confocal microscopy studies revealed a strong correlation between microsecond-scale bubble oscillations and second-to-minute-scale macromolecule diffusion through transient pores formed in the plasma membrane [257]. Although not as prominent, the increased macromolecule uptake was also observed without any additional microbubbles [258,259,260]. This effect was attributed to cavitation caused by the gas bodies present in a medium or acoustic streaming and secondary shear forces [253,261,262]. Introducing microbubbles as cavitation nuclei enhances acoustic energy absorption and lowers the cavitation threshold, thereby intensifying the cavitation-induced bioeffects [263,264,265]. Currently, microbubbles of different sizes, gas filing, and shell composition are commercially available [266].

### 8.1. In Vitro Research on Sonoporation

Extensive research has been conducted since the very first application of ultrasounds in vitro. Nonetheless, due to high variability between study parameters (such as ultrasound frequency, intensity, pressure, presence of cavitation nuclei, size, and composition), it is exceptionally difficult to compare their results. Most of the studies use high-frequency ultrasounds (above 1 MHz), as these are characteristic for therapeutic US transducers (1 to 3 MHz) and diagnostic US transducers (3 to 18 MHz). However, currently, more and more studies also apply low-frequency ultrasound transducers (below 500 kHz) [267]. While high-frequency and high-intensity US leads to less-controllable effects such as heating [268], the effectiveness of low-intensity treatments is often determined by non-thermal effects such as acoustic cavitation and biological signaling [269]. Importantly, using lower frequencies may also provide excessive cellular damage due to the erosive effects of highly energetic inertial cavitation [270]. Both high- and low-frequency ultrasounds have been shown to increase the intracellular transport of fluorescent dyes [271,272,273], genes [274,275,276], and chemotherapeutic drugs [275,276,277,278]. The last one demonstrates the great potential of this technique as an anticancer treatment. Up to now, the efficacy of ultrasound as an enhanced delivery system was proven in various cancer models in vitro, including prostate cancer [273], retinoblastoma [278], squamous carcinoma [279], breast cancer [280], ovarian and liver tumors [281]. The efficacy of sonoporation depends on cell type, hence optimizing the exposure parameters can safely and efficiently increase the cell membrane permeability [281].

### 8.2. Preclinical Studies on Sonoporation

In vitro studies demonstrated the great potential of sonoporation as an anticancer technique. However, these results cannot be directly translated into clinical practice, as the conditions are very different from the actual tumor environment. Among the key factors influencing the effectiveness of sonoporation that are difficult or impossible to mirror in vitro, the following should be mentioned: viscoelasticity of surrounding media and tissue, the number of available cavitation nuclei, the distribution of the ultrasound wave, and finally, the organism immunological response. Regardless of these differences, sonoporation was found to be effective in xenograft models of human epidermoid and human pancreatic carcinoma [282,283], prostate cancer [284], and breast tumors [285]. Especially encouraging was the impact of ultrasounds on improving drug targeting of tumors with low enhanced permeability and retention effect [282]. For that reason, sonoporation is of particular interest in therapies targeting pancreatic cancer. In preclinical study gemcitabine therapy combined with sonoporation significantly impeded tumor development in the orthotopic xenograft model of human pancreatic cancer [283].

### 8.3. Clinical Application of Sonoporation in Oncology

Sonoporation similarly to electroporation can be implemented in the cancer treatment either as a measure for tumor ablation or to evoke increased tumor permeability to anticancer agents. Sonoporation is not targeted at specific cellular mechanisms but rather at producing a biological effect through a generation of physical forces acting on cells. Therefore, its efficacy is much less dependent on the histological type of a tumor compared to non-physical treatment methods.

The most common clinical application of ultrasounds in the context of cancer therapy is high-intensity focused ultrasound (HIFU), which found application in the treatment of various tumors. Some of them, such as ablation of hepatic and breast tumors, are not approved by Food and Drug Administration (FDA), while some are already FDA-approved these include ablation of uterine leiomyomas, ablation of bone metastasis, ablation of prostate cancer, and neurological applications [286]. Extensive studies were carried out using the extracorporeal HIFU device to treat patients with advanced liver cancer, resulting in tumor ablation, palliation, and improved overall survival [287,288,289,290]. Early clinical safety studies were performed on patients with advanced renal tumors suggesting the safety and feasibility of HIFU in this tumor [291]. This technique found application also for the treatment of prostate carcinoma and was proven to be effective as focal therapy for nonmetastatic prostate cancer. However, complications were also reported following HIFU, including urinary retention, incontinence, urinary infection, impotence, chronic pain, rectal and anal fistulas, and burns [287,292]. Ultrasound, microbubbles, and chemotherapy with gemcitabine were safely combined in Phase I of the clinical trial in inoperable pancreatic cancer patients [267]. In the study, sonoporation improved the clinical efficacy of gemcitabine, prolonged the quality of life, and extended survival in patients with pancreatic ductal adenocarcinoma (from 8.9 to 17.6 months, n = 10) without any additional toxicity.

Although promising, current trials demonstrate that ultrasound application also carries the risk of occasionally severe side effects. Miller et al. highlighted several safety factors that still need to be addressed when using sonoporation to achieve the optimum benefit to risk ratio, namely operator and patient safety, quality assurance, accumulating biological effect, risk-benefit ratio, and safety research [293]. Depending on the ultrasound settings, various effects may be evoked in tumors, starting from local permeabilization for targeted drug delivery to extensive tumor destruction by high-frequency and high-intensity ultrasounds. Therefore, there is a considerable need to introduce universal and international operating procedures allowing for a safe and controllable sonoporation application in clinical practice.

## 9. Gravitational Forces Affecting Biomembranes

In recent years, we have witnessed a rapid development of space sciences, which provided us with a plethora of medical and biological challenges concerning life existence and survival in space. Whereas outer space is full of hazardous factors dangerous for man, one of them, namely unnatural gravity—micro- and hyper-gravity—affects cells in a fascinating way leading to altered human physiology. Microgravity is defined as a state where objects have a reduced weight, which is mostly associated with spaceflight. On the other hand, hyper-gravity is a phenomenon mainly experienced during accelerations accompanying spaceship launch, when the gravity force is higher than that on the surface of the Earth [294]. Thus, gravity-related research is a new tool for understanding cancer cell biology, which may help us detect novel molecular targets for future tumor treatment [295,296,297]. The aim of such experiments is to widen current knowledge of cancer treatment in altered gravity and shed new insight on the implementation of gravity-related therapies in medicine in the future. Gravity-based studies constitute an excellent initial step toward enhancing our understanding of the relationship between cellular resistance to chemotherapy and the response to various gravitational stimuli. Going further, the exposure to altered gravity environment by the appropriate combination with chemotherapeutic protocols may become a new agent used in cancer treatment which will allow its medical application in the future.

Until now, a number of studies were carried out under simulated conditions of unnatural gravity using ground-based facilities such as clinostats, random positioning machines, bioreactors, and parabolic flights for simulated microgravity (*sμg*) research [298,299,300], and well-designed centrifuges for hyper-gravity experiments [301,302]. Some real microgravity (*μg*) investigations were conducted in space on the International Space Station or onboard sounding rockets [303]. Both micro- and hyper-gravity was shown to affect various cellular processes such as gene expression [304,305], proliferation [306], differentiation [307,308,309,310,311,312], autophagy [310,313,314] and cell death [305,310,315]. Although the mechanism of cellular graviperception remains unclear [316,317], previous studies suggested an essential role of the cytoskeleton [305], adhesive molecules [318], and cell membrane mechanoreceptors in that process. It is known that the reorganization of cytoskeletal fibers caused by mechanical forces such as altered gravity strongly alters cell functioning [294,319,320]. This phenomenon results in characteristic changes to cell morphology such as the rounded shape of cells, the presence of membrane blebbing [318] as well as the formation of complex 3D spheroid structures [300], or altered functioning of filopodia and lamellipodia [319], which may lead to the reduced migration observed under hyper-gravity [306]. Additionally, there is an interesting connection between cytoskeleton and multidrug resistance (MDR) proteins. Cytoskeletal fibers influence the activity of MDR proteins located in lipid rafts without in gene expression [321]. At the same time, hyper-gravity is thought to affect cell membrane architecture by lipid rafts and its interactions with cytoskeleton [322] and, thus, modify drug sensitivity. Moreover, it was revealed that cells cultured under hyper-gravity display enriched cell membranes with extracellular matrix proteins [323]. Furthermore, this phenomenon was reported to inhibit metastasis and drug resistance of cancer cells [324].

**Figure 5:**
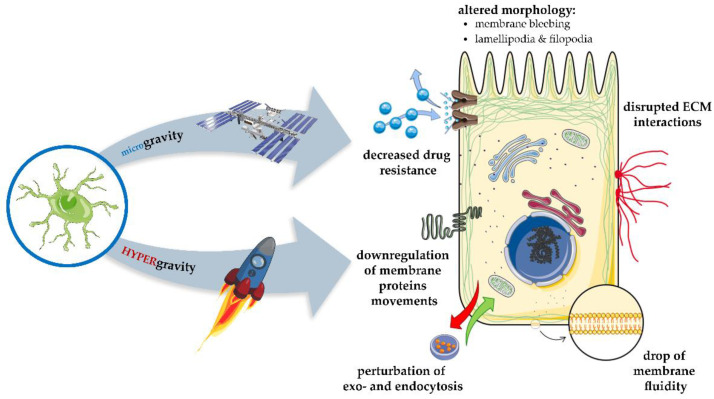
Modification of cell membrane by micro- and hyper-gravity. Altered gravity is known to affect biomembranes on many levels. First, it influences on the morphology of cell by creating membrane blebbing and affects functioning of lamellipodia and filopodia. Furthermore, unnatural gravity alter membrane fluidity and movements of transmembrane proteins leading to decreased multidrug resistance and disruption of exo- and endo-cytosis. This figure was prepared using Servier Medical Art, available from www.servier.com/Powerpoint-image-bank.

A study by Janmaleki et al. revealed that exposure to *sμg* caused a notable drop in cell stiffness and cell membrane viscosity [325]. On the other hand, membrane viscosity affects diffusion-controlled reactions [326] and regulates protein movement within the plasma membrane and cytoplasm [327,328], which alters signaling pathways and gene expression. Furthermore, membrane fluidity depends on gravity loading [329,330,331], which most likely affects pharmacodynamics and drug uptake under unnatural gravity conditions [330]. In light of the above findings, both micro- and hyper-gravity may regulate cell membrane biophysical properties and affect cell functioning on a genetic and proteomic level (Figure 5).

Further research should consider the link between cell membrane, cytoskeleton, and MDR-related proteins and possible molecular pathways proving interactions between them.

## 10. Conclusions

The cell membrane is an exceptionally organized and complex cell element responsible for maintaining the cell structure and interacting with the environment. The possibility of manipulating its composition and function is a powerful tool in cancer treatment, both in combination with chemotherapeutic agents and physical membrane modification methods. In Table 1, we have gathered the types of plasma membrane modifications for targeted cancer therapies, which have been described in the review. The cell membrane and its components must be considered key factors in cancer treatment and deserve consideration when developing new therapeutic strategies. However, it should be kept in mind that the choice of an appropriate cell membrane modification strategy depends on the tumor type, location, and stage.

## Figures and Tables

**Table 1:** Types of plasma membrane modification for targeted cancer therapy.

Method	Type of Membrane Modification	Expected Outcomes	Literature
MDR modulators	Translocation of the ABC superfamily proteins Decrease in the level of ABC superfamily proteins Altered lipid integrity, fluidity or permeability of cell membrane Modulation of ABC superfamily proteins activity	Increase in cellular sensitivity to anti-cancer drugs/decrease of MDR	[35] [48] [52,54,55] [31,34,41,47,49,50,51,52,53,56,57,58,59,60,61,62,63,64]
MicroRNAs as regulators of MDR	Decrease in the level of ABC superfamily proteins	Increase in cellular sensitivity to anti-cancer drugs/decrease of MDR	[74,75,76,77,78,79,80,81,82,83,84,85,86,87]
Modification of IC	Diminish of IC activity by natural and synthetic inhibitors IC inhibition by antibodies IC expression decrease by siRNA, miRNA, CRISPR/Cas9	Alterations in ion efflux/influx; inhibition of cell proliferation, motility, and invasiveness; increase of cell apoptosis and sensitivity for anticancer drugs	[106,111,113,116,122] [127,128] [132,133,134,135]
Membrane receptors modulations	Inhibition of membrane receptors	Sensitize cancer cells to conventional therapy	[155,156,157,158,159,160,164,165,166,167,168,169]
Membrane lipidomics modulations	Changes in the composition of membrane lipids Changes in the activity of membrane-related enzymes and signaling pathways	Increased membrane permeability, decreased drug resistance Sensitize cancer cells to conventional therapy	[3,171,172,173,175,176,178,179,181,182,185,186] [177,186]
Electroporation	Induction of pores in the lipid membrane. Irreversible pores-membrane disruption	Increased membrane permeability/cell lysis, delivering drugs into the cell	[188,189,190,191,192,195,202,203,204,205,206,220,221,222,223,224,225,229,230,231,233,234,235,236,238,239,242,243]
Sonoporation	Membrane invaginations—pores, endocytotic vesicles; membrane disruption	Increased membrane permeability/cell lysis, delivering drugs into the cell	[252,253,268,270]
Gravitational forces	Membrane blebbing; drop of membrane fluidity; disrupted ECM interactions and membrane proteins movements	Decreased drug resistance; altered cell morphology	[294,295,296,297,298,299]

## Data Availability

Data sharing not applicable. No new data were created or analyzed in this study. Data sharing is not applicable to this article.
